# Supplementary material for: Unexpected arousal modulates the influence of sensory noise on confidence
Source: eLife. 2016 Oct 25;5:e18103. doi: 10.7554/eLife.18103 (PMC5079750; doi:10.7554/eLife.18103)
Supplement: Figure 2—source data 2. — Please see Materials and methods for full descriptions of all variables. DOI: http://dx.doi.org/10.7554/eLife.18103.005 [file elife-18103-fig2-data2.docx]

**Source files for behavioural results (related to figure 2).**

**Figure 2-source data 1.**

This csv table contains the data for Figure 2. All data are split by condition, NL = “neutral cue low variance”, NH = “Neutral cue high variance”, DL = “disgust cue low variance, DH = “disgust cue high variance”.

**Figure 2- source data 2.**

Table with variable codes used in Figure 2-source data 1. Please see Methods for full descriptions of all variables.

| **Code** | **Explanation** |
| --- | --- |
| medianRT | Median Reaction Time |
| meanAcc | Average Accuracy (hit rate) |
| medianOrr | Median signal orientation (degrees) |
| da | D-prime (from SDT model) |
| mr | M-ratio (from SDT model) |
| mca | Type-I criterion (from SDT model) |
| T2ca_all | Type-II Criterion (from SDT model) |
| F1-F4_dprime | Force-choice d-prime for each of the four face primes. |
